# β-Lapachone suppresses the lung metastasis of melanoma via the MAPK signaling pathway

**DOI:** 10.1371/journal.pone.0176937

**Published:** 2017-05-08

**Authors:** Ji-Ye Kee, Yo-Han Han, Dae-Seung Kim, Jeong-Geon Mun, Seong-Hwan Park, Hong-Seob So, Sung-Joo Park, Raekil Park, Jae-Young Um, Seung-Heon Hong

**Affiliations:** 1 Department of Oriental Pharmacy, College of Pharmacy, Wonkwang-Oriental Medicines Research Institute, Wonkwang University, Iksan, Jeonbuk, Republic of Korea; 2 Center for Metabolic Function Regulation, Wonkwang University, Iksan, Republic of Korea; 3 Department of Herbology, College of Oriental Medicine Wonkwang University, Iksan, Jeonbuk, Republic of Korea; 4 Department of Biomedical Science and Engineering, Gwangju Institute of Science and Technology (GIST), Gwangu, Republic of Korea; 5 Department of Pharmacology, College of Korean Medicine, Institute of Korean Medicine, Kyung Hee University, Dongdaemun-gu, Seoul, Republic of Korea; University of South Alabama Mitchell Cancer Institute, UNITED STATES

## Abstract

β-Lapachone is a natural quinone compound from Lapacho trees, which has various pharmacological effects such as anti-bacterial, anti-fungal, anti-viral, and anti-inflammatory activities. However, the effect of β-lapachone on metastasis of melanoma cells is unclear. In this study, β-lapachone reduced cell viability of metastatic melanoma cancer cell lines B16F10 and B16BL6 through induction of apoptosis via the mitogen-activated protein kinase (MAPK) pathway. Additionally, flow cytometry results showed that β-lapachone increased DNA content in the G0/G1 phase of the cell cycle. Analysis of the mechanisms of these events indicated that β-lapachone regulated the expression of Bcl-2, Bcl-xL, and Bax, resulting in the activation of caspase-3, -8, -9, and poly-ADP-ribose polymerase (PARP). Moreover, the β-lapachone-administered group showed significantly decreased lung metastasis in the experimental mouse model. In conclusion, our study demonstrates the inhibitory effect of β-lapachone on lung metastasis of melanoma cells and provides a new insight into the role of β-lapachone as a potential antitumor agent.

## Introduction

Melanoma is a highly aggressive cancer and is the leading cause of death from skin cancer because of its resistance against most conventional treatments and tendency to metastasize [1]. Worldwide statistics show that melanoma incidence and mortality rates have been increasing for at least 30 years [2]. In addition, the prognosis for melanoma remains very poor, with a 5-year survival rate of less than 5% [3,4]. The most dangerous aspect of melanoma is its metastatic ability to spread to other organs such as the liver, lungs, brain, and bones in later stages [5]. Therefore, new effective and safe therapeutic agents for metastatic melanoma are needed.

Metastasis is caused by movement of cancer cells from the primary tumor to target organs. Thus, cancer cell migration and invasion abilities are associated with metastasis. Epithelial-to-mesenchymal transition (EMT) is thought to be an important mechanism for promoting cancer progression through the induction of cancer cell migration and invasion. EMT is the loss of epithelial characteristics and acquisition of mesenchymal morphology. The downregulation of the epithelial protein E-cadherin and up-regulation of mesenchymal proteins including N-cadherin and vimentin are considered a hallmark of EMT [6–8]. Matrix metalloproteinases (MMPs) such as MMP-2 and MMP-9 play critical roles in the proteolytic degradation of the extracellular matrix (ECM) surrounding the primary tumor, which is required for the migration and invasion of cancer cells [9]. Inhibition of MMP-2 and MMP-9 expression and activity in cancer cells has been shown to prevent their migration and invasion.

Cancer cells represent several differences compared to normal cells including uncontrolled cell proliferation, and mutation of specific genes. The cell cycle is regulated by the cyclins which are the regulatory proteins and cyclin-dependent kinases (CDKs). Overexpression of cyclins and CDKs leads to dysregulation of the cell cycle in cancer cells [10]. When cancer cells are damaged to DNA, cell cycle is arrested to repair. However, failure of DNA repair causes to cell cycle arrest proceeds apoptosis [11]. Apoptosis is known as programmed cell death and it occurred to maintain the homeostasis through extrinsic and intrinsic pathways. Morphological features of apoptosis are nuclear fragmentation and chromatin condensation in the nucleus as well as cell shrinkage and irregularities in shape. Apoptosis is progressed without noticeable symptoms such as release of inflammatory factors [12]. Therefore, induction of apoptosis and cell cycle arrest is the efficient method for cancer treatment.

β-Lapachone is a natural quinone compound derived from the lapacho tree (*Tabebuia avellanedae*). It is used as herbal medicine by indigenous people in South and Central America. This compound has various pharmacological effects including anti-bacterial, anti-viral, and anti-inflammatory activities [13–15]. In addition, β-lapachone exhibited an anti-cancer effect in various types of cancer, including liver, breast, prostate, and colon cancer [16–19]. In particular, it induced cytotoxicity in melanoma cells through reactive oxygen species, and NAD(P)H:quinone acceptor oxidoreductase (NQO) 1-mediated redox activation [20]. However, the anti-metastatic effects of β-lapachone on melanoma cells were rarely reported. Therefore, β-lapachone-induced inhibitory effects on the metastatic ability of melanoma cells were investigated in this study.

## Materials and methods

### Reagents

Anti-phospho-p38, anti-phospho-ERK, anti-phospho-JNK, PARP, caspase-3, caspase-8, and antibodies were purchased from Cell Signaling Technology, Inc. (Danvers, MA, USA). Caspase-9 and ERK antibodies were obtained from Enzo Life Sciences (Farmingdale, NY, USA). Bcl-2, p38, JNK, and GAPDH antibodies were purchased from Santa Cruz Biotechnology, Inc. (Santa cruz, CA, USA). Bcl-xL and Bax antibodies were purchased from Bioworld Technology (Louis Park, MN, USA). Transwell chamber and matrigel were obtained from BD Biosciences (San Diego, CA, USA). β-Lapachone was chemically synthesized by KT&G Life Science (Suwon, Korea).

### Cell culture

The mouse melanoma cell lines B16F10 and B16BL6 were purchased from Korean cell line bank (Seoul, Republic of Korea) and cultured in Dulbecco's Modified Eagle's Medium (DMEM; Gibco BRL, Grand Island, NY, USA) supplemented with 10% fetal bovine serum (FBS), 100 units/ml penicillin, and 100 μg/ml streptomycin at 37°C in a 5% CO_2_ incubator. During the experiment, mycoplasma was controlled by Mycoplasma Removal Agent (MP Biomedicals, Solon, OH, USA).

### Animals

C57BL/6 female mice (5 weeks old, 19–20 g) were purchased from Samtaco Korea (Osan, Korea). The animals were cared in a laminar air-flow room maintained at a temperature of 22 ± 1°C with a 12 h light and 12 h dark circle. The mice were sacrificed by diethyl ether inhalation when abnormal status such as body weight was reduced by 10% of normal weight was observed.

### WST assay

Cell viability was measured using the WST-8 [2-(2-methoxy-4-nitrophenyl)-3-(4-nitrophenyl)-5-(2, 4-disulfophenyl)-2H-tetrazolium, monosodium salt] reagent (Enzo Life Sciences, Farmingdale, NY, USA). Cells (5 × 10^3^ cells/well) were seeded in 96-well microplates and β-lapachone was added to each well. After 24–72 h of incubation, the medium was changed to WST-8 reagent with medium, and absorbance was measured at 450 nm using a microplate reader (Molecular Devices, Sunnyvale, CA, USA).

### Cell cycle analysis

Cell cycle analysis was carried out using the Muse Cell Cycle Kit (Millipore, Bedford, MA, USA) according to the manufacturer's instructions. B16F10 cells (1 × 10^6^ cells) were seeded in 6-well plates and incubated overnight. The cells were treated with β-lapachone for 48 h and harvested. They were then fixed with 70% ice-cold ethanol at -20°C for at least 3 h and washed with PBS. Cell pellets were resuspended in 100 μl of Cell Cycle Reagent and incubated for 30 min at room temperature in the dark. Cells were analyzed using Muse Cell Analyzer and the cell cycle phase distribution was quantified using Muse analysis software (MUSE, Millipore, Bedford, MA, USA).

### TUNEL assay

A terminal nucleotidyl transferase-mediated nick end labeling (TUNEL) assay was carried out using In Situ Cell Death Detection Kit, Fluorescein (Roche Diagnostics GmbH, Penzberg, Germany) according to the manufacturer’s protocol. Cells were seeded in 8-well chamber slide and treated with β-lapachone. After 24 h, the cells were fixed in 3.7% formaldehyde at room temperature (RT) for 1 h, and permeabilized in 0.1% Triton X-100 at ice for 2 min. After washing with PBS, cells were incubated with the TUNEL reaction mixture for 1 h at 37°C, and stained with DAPI (2 μg/ml). TUNEL positive cells were observed and images were photographed by the Zeiss Observer A1 microscope (Carl Zeiss, Oberkochen, Germany).

### Annexin V assay

Annexin V assay was conducted using the MUSE Annexin V and dead cell kit in accordance with the recommended protocol (Millipore, Billerica, USA). Briefly, B16F10 cells (1 × 10^6^ cells) were resuspended in 100 μl of culture medium containing 1% FBS and incubated with 100 μl of Muse Annexin V and Dead Cell Reagent which contained annexin V and 7-aminoactinomycin D (7-AAD), for 20 min at room temperature in the dark. The cells were quantified using the Muse Cell Analyzer and Muse analysis software.

### Western blot analysis

B16F10 cells (1 × 10^6^ cells/well) were treated with various concentrations of β-Lapachone and lysed in PRO-PREP^™^ Protein Extraction solution (iNtRon Biotech, Seoul, Korea) for 1 h. Cell lysates were centrifuged for 10 min and the supernatant was mixed with 2X sample buffer. Samples were separated by sodium dodecyl sulfate-polyacrylamide gel electrophoresis (SDS-PAGE) and were transferred to an Immobilon-P nylon membrane. The membranes were blocked with 5% skim milk for 1 h 30 min and incubated for 3 h with primary antibodies. These antibodies were detected using horseradish peroxidase (HRP)-conjugated secondary antibodies and bands were detected using the ECL system (GE Healthcare UK, Buckinghamshire, UK).

### Real-time RT-PCR

Total RNA was extracted from cells using an RNA-spin^™^ Total RNA Extraction Kit (iNtRon Biotech, Seoul, Korea) according to the manufacturer’s instructions. Total RNA (2 μg) was reverse-transcribed and amplified by PCR using oligo(dT)18 primer and Power cDNA Synthesis Kit (iNtRon Biotech, Seoul, Korea). Reverse transcription was performed at 42°C for 50 min and then at 70°C for 15 min. Real-time quantitative RT-PCR was performed using a Power SYBR^®^ Green PCR Master Mix and step-one plus^™^ real-time pcr systems (Applied Biosystems, Foster City, CA, USA). Values were normalized to GAPDH mRNA. Primer sequences are summarized in Table 1.

**Table 1:** Sequences of real-time RT-PCR primers.

Gene	Forward (5′-3′)	Reverse (5′-3′)
MMP-2	CCCCATGAAGCCTTGTTTACC	TTGTAGGAGGTGCCCTGGAA
MMP-9	AGACCAAGGGTACAGCCTGTTC	GGCACGCTGGAATGATCTAAG
E-cadherin	AATGGCGGCAATGCAATCCCAAGA	TGCCACAGACCGATTGTGGAGATA
N-cadherin	TGGAGAACCCCATTGACATT	TGATCCCTCAGGAACTGTCC
Vimentin	CGGAAAGTGGAATCCTTGCA	CACATCGATCTGGACATGCTG
Snail	TCCAAACCCACTCGGATGTGAAGA	TTGGTGCTTGTGGAGCAAGGACAT
Slug	CATTGCCTTGTGTCTGCAAG	AGAAAGGCTTTTCCCCAGTG
Twist	AGCTACGCCTTCTCCGTCT	TCCTTCTCTGGAAACAATGACA
Cyclin D1	TAGGCCCTCAGCCTCACTC	CCACCCCTGGGATAAAGCAC
CDK4	AGAGCTCTTAGCCGAGCGTA	TTCAGCCACGGGTTCATATC
p16	AATCTCCGCGAGGAAAGC	GTCTGCAGCGGACTCCAT
GAPDH	GACATGCCGCCTGGAGAAAC	AGCCCAGGATGCCCTTTAGT

### Wound healing assay

B16F10 cells were seeded in a 6-well plate (1 × 10^6^ cells/well) and incubated to 8–90% confluence. Scratch was made using a 200 μl micropipet tip and changed to serum-free medium with or without various concentrations of β-lapachone. Mitomycin c (Sigma, St Louis, MO, USA) was added to suppress the cell proliferation. Images were taken at 0 h and 24 h with phase contrast microscopy (Leica, Wetzlar, Germany).

### Invasion assay

The invasion ability of B16F10 cells was measured with a Boyden chamber invasion assay. The inner part of the transwell chamber was precoated with matrigel overnight. Cells (5 × 10^4^ cells/ml) were seeded in serum-free medium with β-lapachone and added to the upper part of the transwell chamber for 24 h. The lower part of the transwell chamber was filled with 10% FBS in DMEM as a chemoattractant. The chambers were washed twice with PBS and fixed in 3.7% paraformaldehyde for 10 min. After being washed twice more with PBS, the fixed cells were treated with 100% methanol for 20 min and stained with Giemsa for 15 min. The inner side of the chambers was wiped with a cotton swab. The membrane inserts were dried and observed under a microscope (Leica, Wetzlar, Germany).

### Gelatin zymography

Cells (5 × 10^5^ cells/well) were seeded in 6-well plates and maintained in serum-free medium for 12 h prior to designated treatments with β-lapachone. After 24 h, the conditioned medium was collected and mixed with 5X sample buffer. Samples were subjected to electrophoresis on an 8% SDS-PAGE gel containing 0.1% gelatin. The gels were washed twice in renaturing buffer (pH 7.5, 2.5% Triton X-100) for 15 min, then incubated in developing buffer (50 mM Tris-HCl pH 7.5, 10 mM CaCl_2_, 0.05% NaN_3_, and 150 mM NaCl) at 37°C for 24 h. The gelatinolytic activity of MMPs was visualized by staining the gels with Coomassie blue R-250 solution and destaining with destaining buffer (50% methanol, 10% acetic acid, and 40% distilled water) for 30 min. The non-stained band caused by the presence of the MMPs was photographed using a digital imaging system.

### Experimental lung metastasis model

Mice were injected intravenously via the tail vein (i.v.) with B16F10 cells (2 × 10^5^ cells) in 200 μl of PBS. Each mouse was administered 50 μl of DMSO as the control or 5 mg/kg β-lapachone by intraperitoneal injection 2 h prior to the injection of B16F10 cells; this treatment was repeated once every 2 days. The condition of the mice was monitored once every 2 days and checked body weight of mice every week. No one died after tumor implantation during the *in vivo* experiment. After 14 days, mice were anaesthetized and sacrificed with diethyl ether inhalation. The lungs were removed and fixed in 3.7% formaldehyde. The number of tumor colonies in the lung was counted to evaluate tumor metastasis. This study was conducted in accordance with the internationally accepted principles for laboratory animal use and care as found in the Wonkwang University Institutional Animal Care and Use Committee (IACUC) guidelines (WKU14-17). This certification specifically approved *in vivo* experiment using lung metastasis mouse model in this study from Wonkwang University IACUC.

### Statistical analysis

Data was analyzed using the Student's t-test for statistical significance. *P*-value < 0.05 was considered statistically significant differences. Data are presented as the mean ± SD of the three experiments.

## Results

### β-Lapachone reduces cell viability of melanoma cells

**Fig 1:**
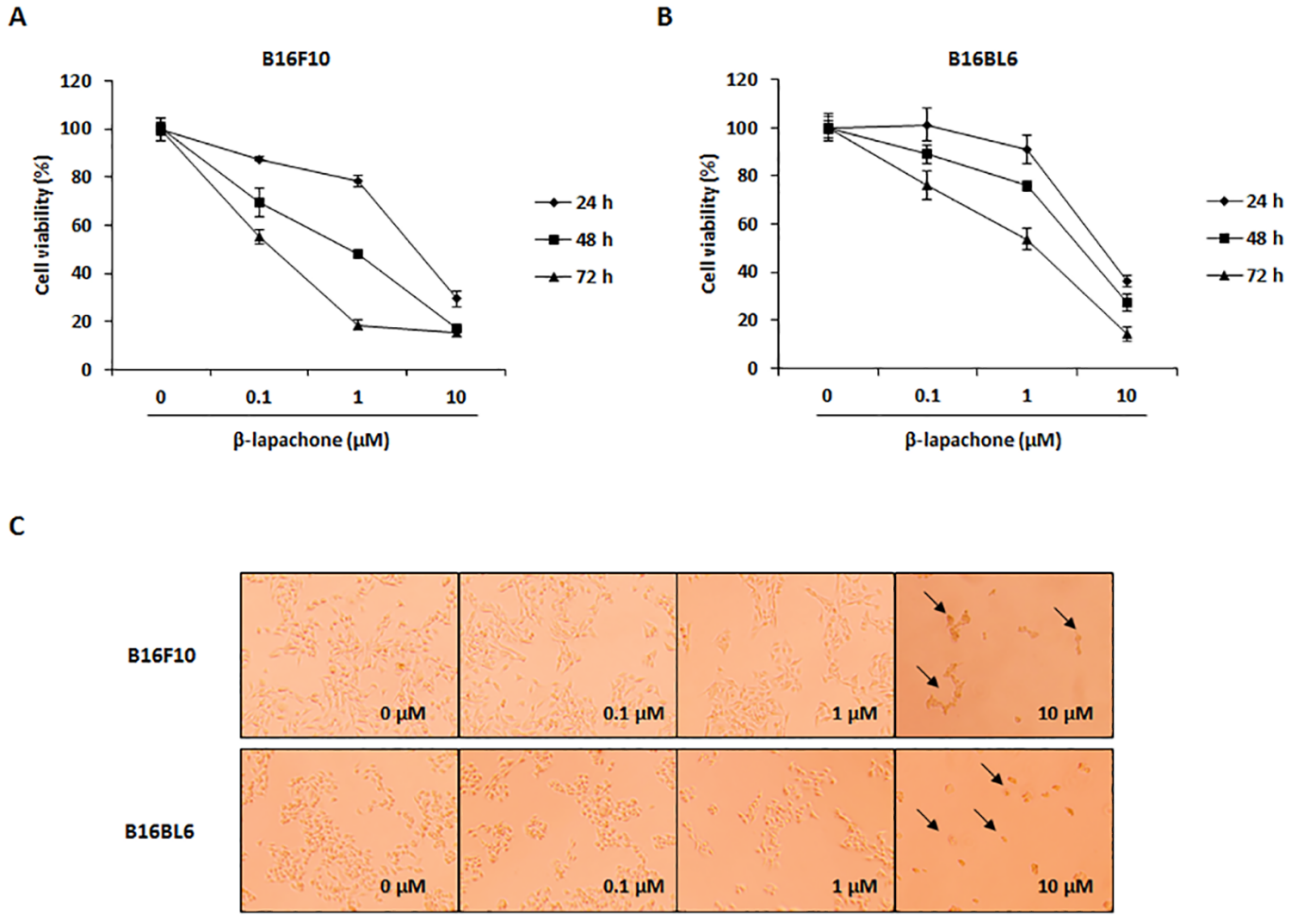
Effect of β-lapachone on the cell viability of metastatic melanoma cells. (A and B) Cell viability of β-lapachone-treated B16F10 (A) and B16BL6 (B) cells. The cells were seeded at a density of 5 × 10^3^ cells/well in 96-well microplates and then treated with various concentrations of β-lapachone for 24–72 h. After incubation, cell viability was determined by WST assay. Values are the means ± SD of data from three independent experiments. (C) Morphology of β-lapachone-treated cells. B16F10 and B16BL6 cells were treated with β-lapachone for 48 h. The morphological changes of the cells were observed under a phase-contrast microscope (magnification 200×).

The effect of β-lapachone on metastatic melanoma cell growth was investigated in B16F10 and B16BL6 cells. Using the WST assay, we found that β-lapachone exhibited cytotoxicity in metastatic melanoma cell lines B16F10 and B16BL6 in a time- and dose-dependent manner (Fig 1A and 1B). In addition, photographs of β-lapachone-treated B16F10 cells showed morphology characteristic of apoptosis, such as reduction in cell number and increased number of rounded cells (Fig 1C). In particular, β-lapachone showed much stronger growth inhibitory effect in B16F10 cells than in B16BL6 cells. Therefore, we selected B16F10 cells for subsequent experiments.

### β-Lapachone induces cell cycle arrest at the G0/G1 phase

**Fig 2:**
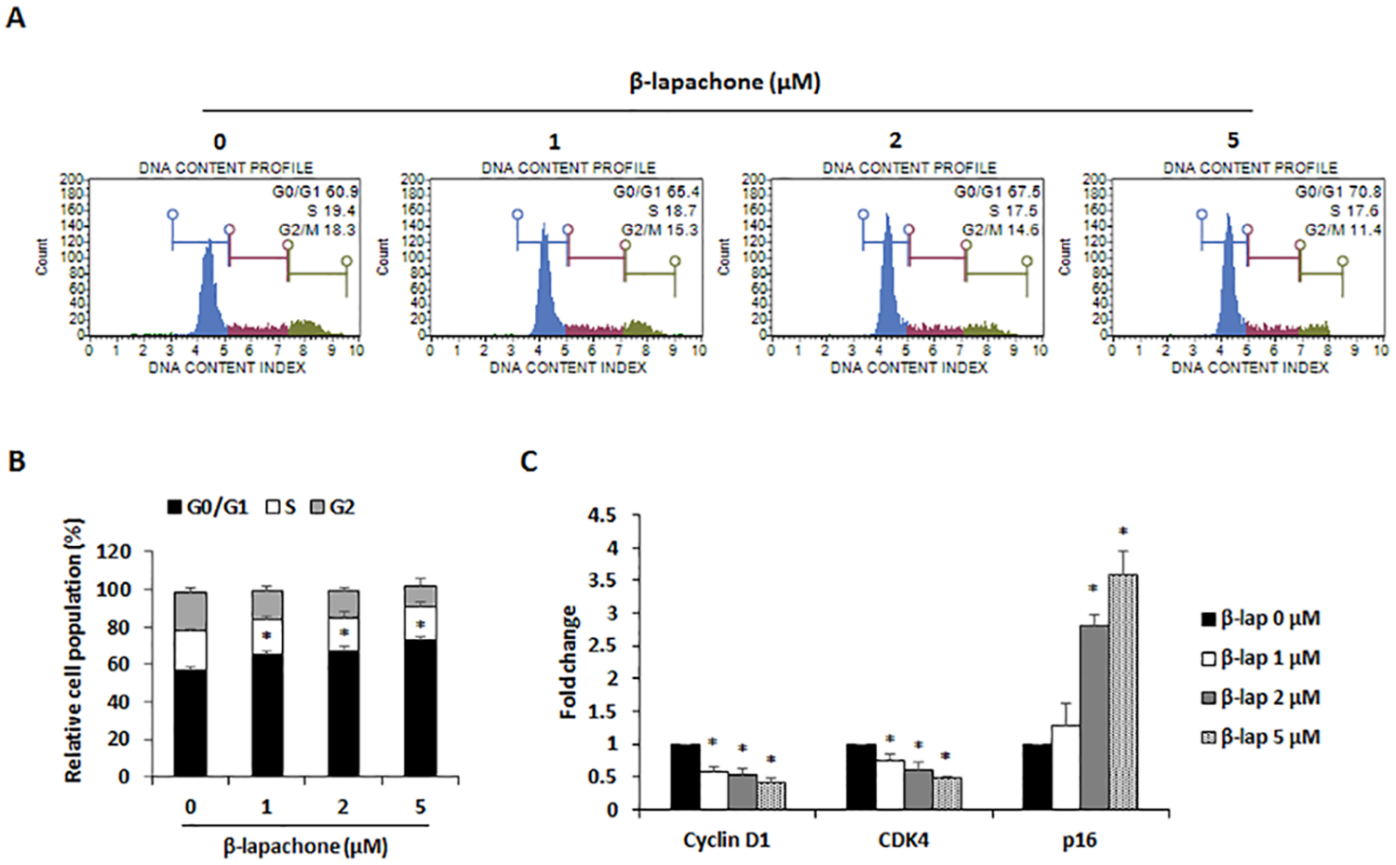
Effect of β-lapachone on cell cycle arrest in B16F10 cells. (A) Cell cycle analysis on β-lapachone-treated B16F10 cells. The cells were treated with β-lapachone (1, 2, and 5 μM) for 48 h and fixed with 70% ethanol for at least 3 h. After centrifugation, cells were stained with PI solution and analyzed for determination of cell cycle phase distribution. Data are representative of three independent experiments. (B) The percentage of cells in each phase of the cell cycle. (C) mRNA expression of cyclin D1 and CDK4. B16F10 cells were treated with indicated concentrations of β-lapachone for 24 h. Values are the means ± SD of data from three independent experiments. *P < 0.05.

To confirm that the inhibitory effect of β-lapachone on melanoma cell growth was partly due to cell cycle arrest, B16F10 cells were treated with various concentrations of β-lapachone for 24 h, and cell cycle analysis was performed using propidium iodide (PI) staining. Treatment with β-lapachone dose-dependently increased the G0/G1 population of B16F10 cells (Fig 2A and 2B). Moreover, significantly lower levels of cyclin D1 and CDK4 expression were observed, whereas inhibitor of the CDK4-Cyclin D1 complex p16 expression was decreased in B16F10 cells treated with β-lapachone (Fig 2C). These results suggested that β-lapachone may regulate the cell cycle of melanoma cells by reducing cyclin D1 and CDK4 expression.

### β-Lapachone induces apoptosis in melanoma cells

**Fig 3:**
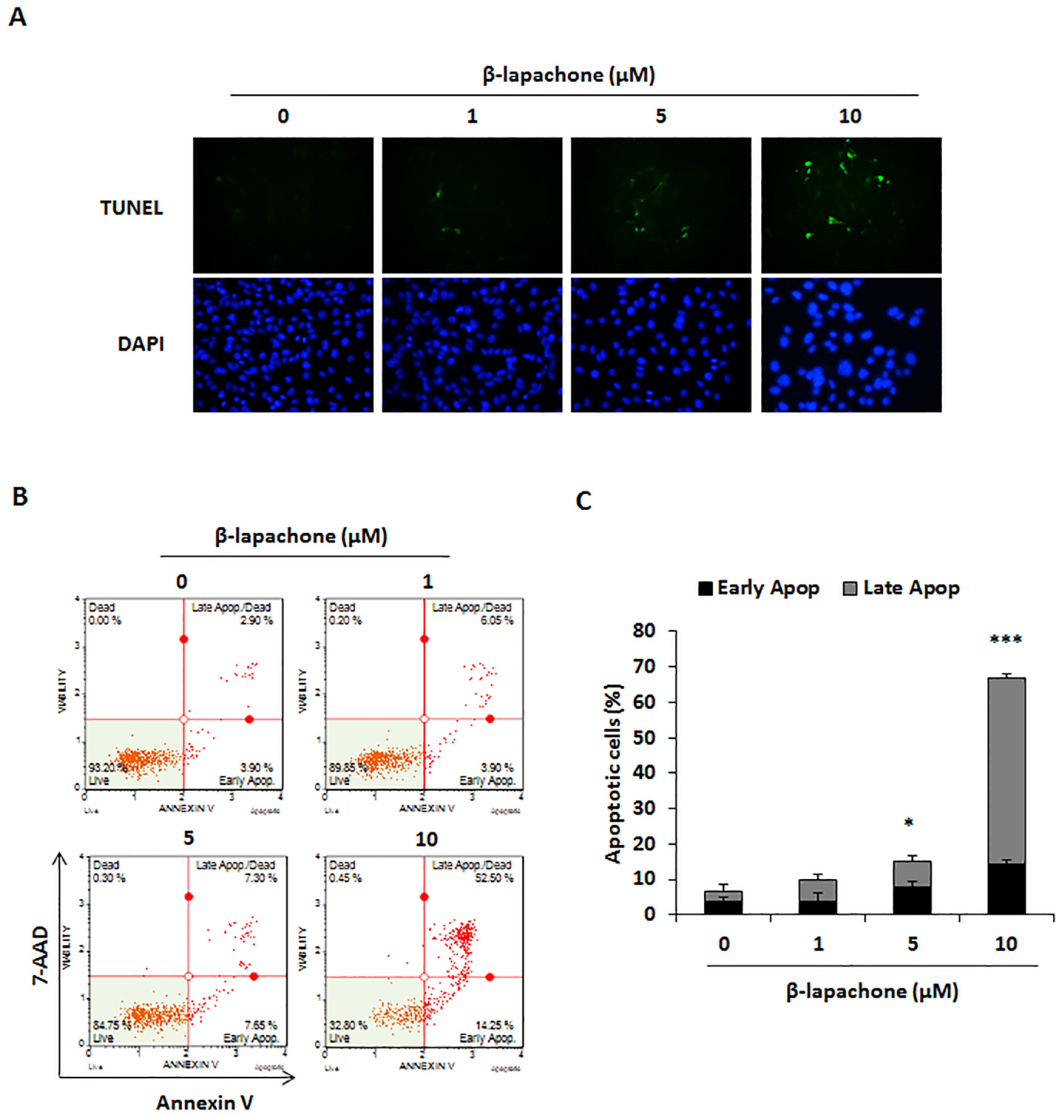
Effect of β-lapachone on the apoptosis of B16F10 cells. (A) β-Lapachone-induced apoptosis was observed by TUNEL assay under an inversion fluorescent microscope. B16F10 cells were incubated with indicated concentration of β-lapachone for 12 h. (B) B16F10 cells were incubated with the indicated concentrations of β-lapachone for 12 h and analyzed by Annexin V/7-AAD staining and flow cytometry. The figure is representative of three independent experiments. (C) The percentages of apoptotic cells were plotted. Values are the means ± SD of data from three independent experiments. *P < 0.05 and ***P < 0.001.

Considering the growth inhibitory effect of β-lapachone on metastatic melanoma cells, we investigated whether β-lapachone induced apoptosis of B16F10 cells. After cells were treated with β-lapachone (5 and 10 μM) for 24 h, increased TUNEL positive cells were observed by TUNEL assay (Fig 3A). To further confirm whether β-lapachone induced apoptosis, B16F10 cells were exposed to β-lapachone for 24 h and analyzed using flow cytometric measurement after Annexin V/7-AAD staining. β-Lapachone markedly induced cell apoptosis of B16F10 cells, as shown by the percentage of apoptotic cells (Fig 3B and 3C).

### β-Lapachone induces apoptosis via MAPK signaling pathway in melanoma cells

**Fig 4:**
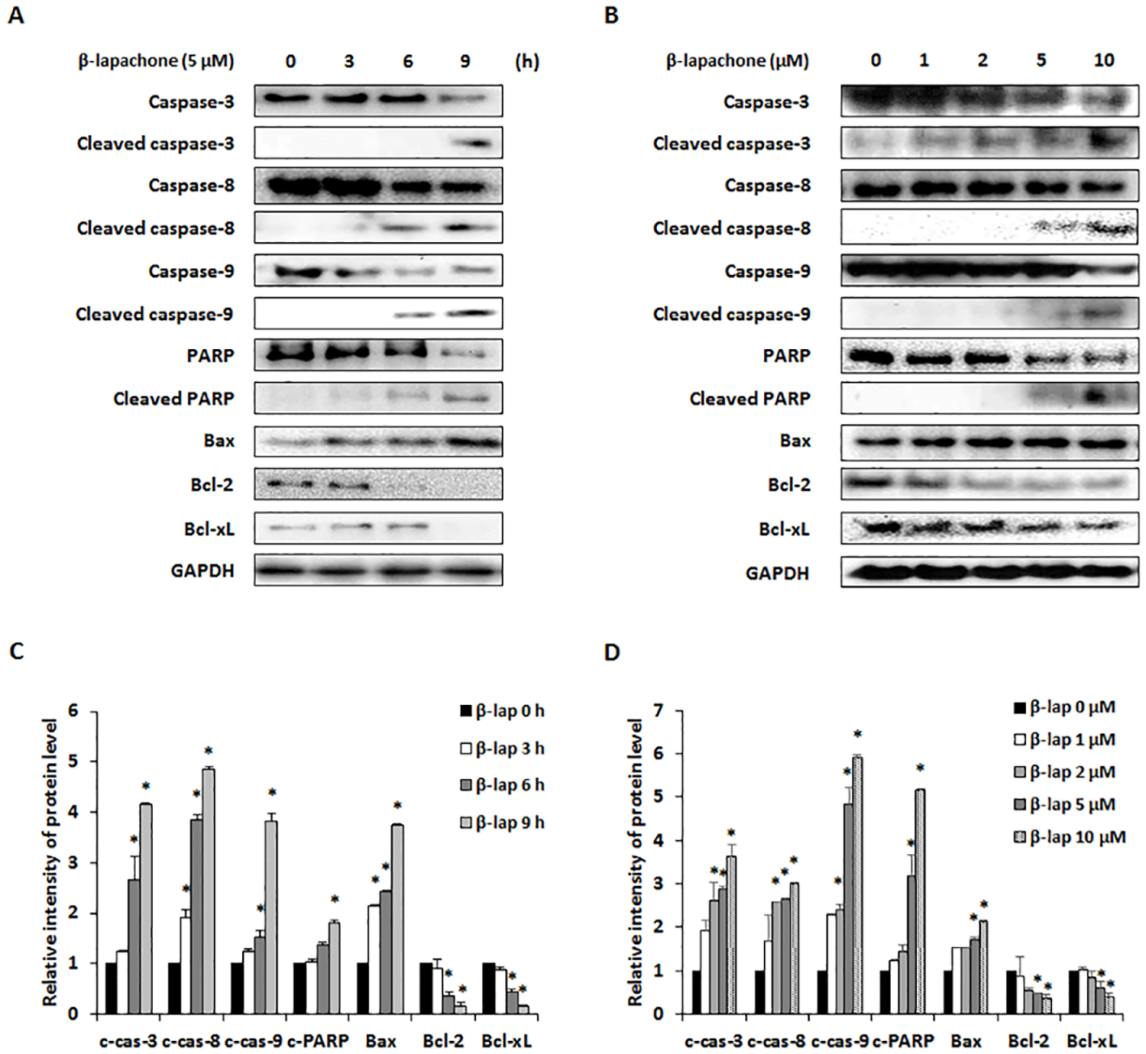
Effect of β-lapachone on the apoptotic pathway in B16F10 cells. A and B. B16F10 cells were treated with β-lapachone (5 μM) for 0–9 h (A) or various concentrations of β-lapachone for 12 h (B). Cell lysates were subjected to western blot analysis with PARP, caspase-3, -8, -9, Bcl-2, Bcl-xL, and Bax antibodies. (C and D) Relative levels of cleaved caspase-3 (c-cas-3), cleaved caspase-8 (c-cas-8), cleaved caspase-9 (c-cas-9), cleaved PARP (c-PARP), Bax, Bcl-2, and Bcl-xL were calculated using an Image J program. Values are the means ± SD of data from three independent experiments. *P < 0.05.

**Fig 5:**
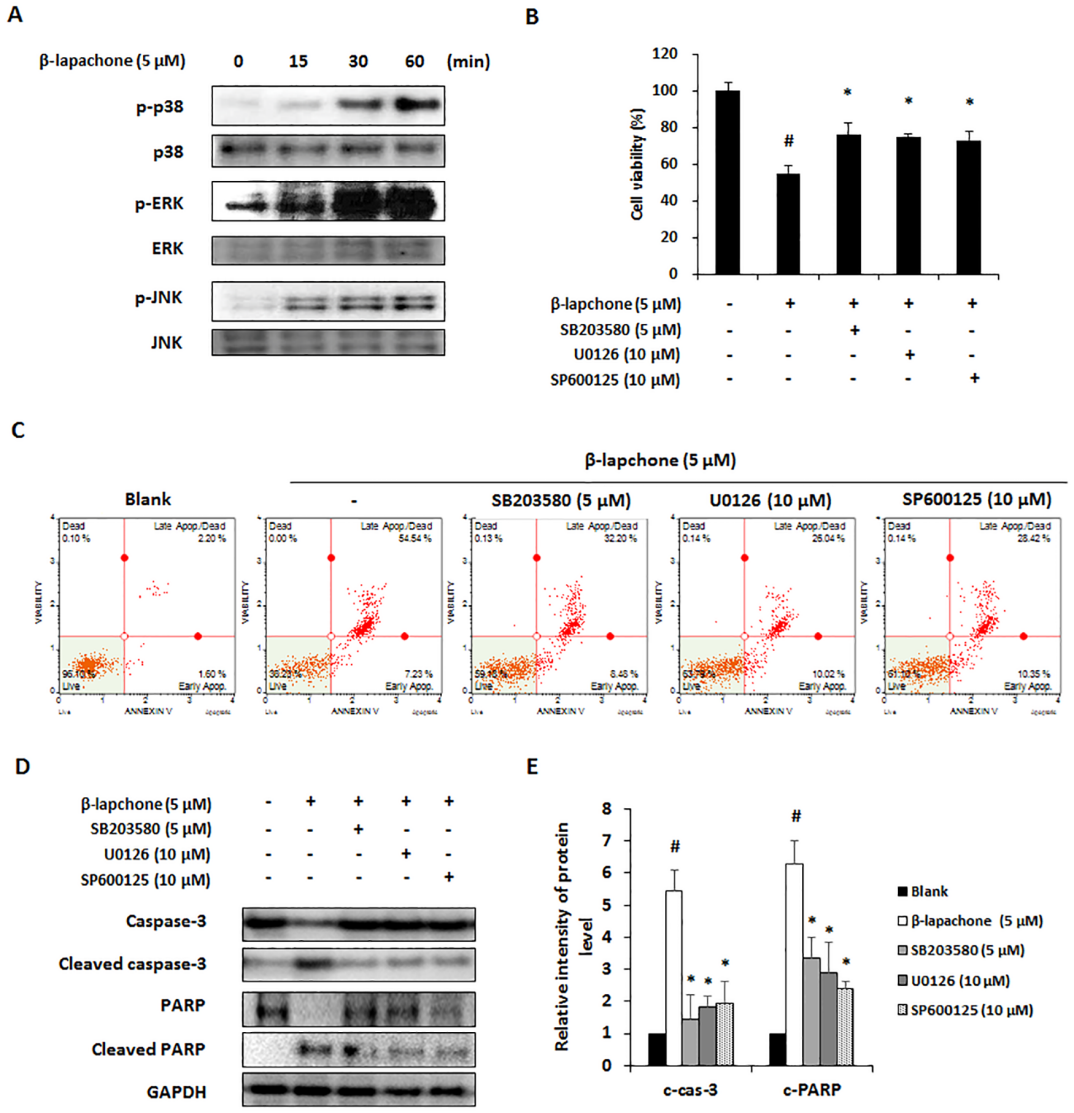
Effect of β-lapachone on the MAPKs pathway in B16F10 cells. (A) B16F10 cells were exposed to β-lapachone (5 μM) for 0–60 min and phosphorylation levels of MAPK proteins were determined by western blot analysis. (B and C) Role of MAPKs signaling on the β-lapachone-induced apoptosis in B16F10 cells. Cells were pretreated with the MAPK inhibitors SB203580 (5 μM, p38 inhibitor), U1026 (10 μM, ERK inhibitor), and SP600125 (10 μM, JNK inhibitor). After β-lapachone treatment for 24 h, cell viability was determined by WST assay (B) and Annexin V assay (C). (D) Expressions of caspase-3 and PARP in the β-lapachone and MAPK inhibitors SB203580, U1026, SP600125-treated B16F10 cells. (E) Relative levels of cleaved caspase-3 (c-cas-3) and cleaved PARP (c-PARP) were calculated using an Image J program. Values are the means ± SD of data from three independent experiments. Values are the means ± SD of data from three independent experiments. ^#^*P* < 0.05 versus β-lapachone non-treated cells, *P < 0.05 versus only β-lapachone-treated cells.

To explore the mechanisms of β-lapachone-induced apoptosis, we evaluated the expression of pro- and anti-apoptotic proteins in B16F10 cells. Western blot data showed that β-lapachone time- and dose-dependently induced pro-apoptotic proteins caspase-3, -8, and -9, PARP cleavage, and increased expression of Bax in B16F10 cells, whereas expression of anti-apoptotic proteins such as Bcl-2 and Bcl-xL was decreased in these cells (Fig 4). MAPKs including p38, ERK, and JNK, are implicated in regulating survival and cell death responses of tumor cells, and play critical roles in the regulation of cell proliferation, differentiation, and apoptosis [21–23]. Thus, we examined whether MAPKs are involved in β-lapachone-induced apoptosis. Western blot analysis data showed that the phosphorylation of p38, ERK, and JNK was increased by β-lapachone in B16F10 cells (Fig 5A). To clarify whether p38, ERK, and JNK were involved in β-lapachone-induced apoptosis, cells were treated with β-lapachone in the presence of SB203580 (p38 inhibitor), U0126 (ERK inhibitor), and SP600125 (JNK inhibitor). As shown in Fig 5B, the inhibition of p38, ERK and JNK phosphorylation significantly improved cell viability of β-lapachone-treated B16F10 cells. Additionally, blockage of p38, ERK and JNK phosphorylation suppressed β-lapachone-induced apoptosis. β-lapachone (5 μM) increased apoptotic cells to 61.77%, whereas co-treatment of SB203580, U0126, and SP600125 decreased β-lapachone-induced apoptosis to 40.68%, 36.06%, and 38.77%, respectively. Moreover, these MAPK inhibitors inhibited cleavage of caspase-3 and PARP by β-lapachone treatment (Fig 5D and 5E). These results demonstrated that β-lapachone induces apoptosis through activation of p38, ERK, and JNK in B16F10 cells.

### β-Lapachone inhibits cancer cell migration and invasion through MMP activity

**Fig 6:**
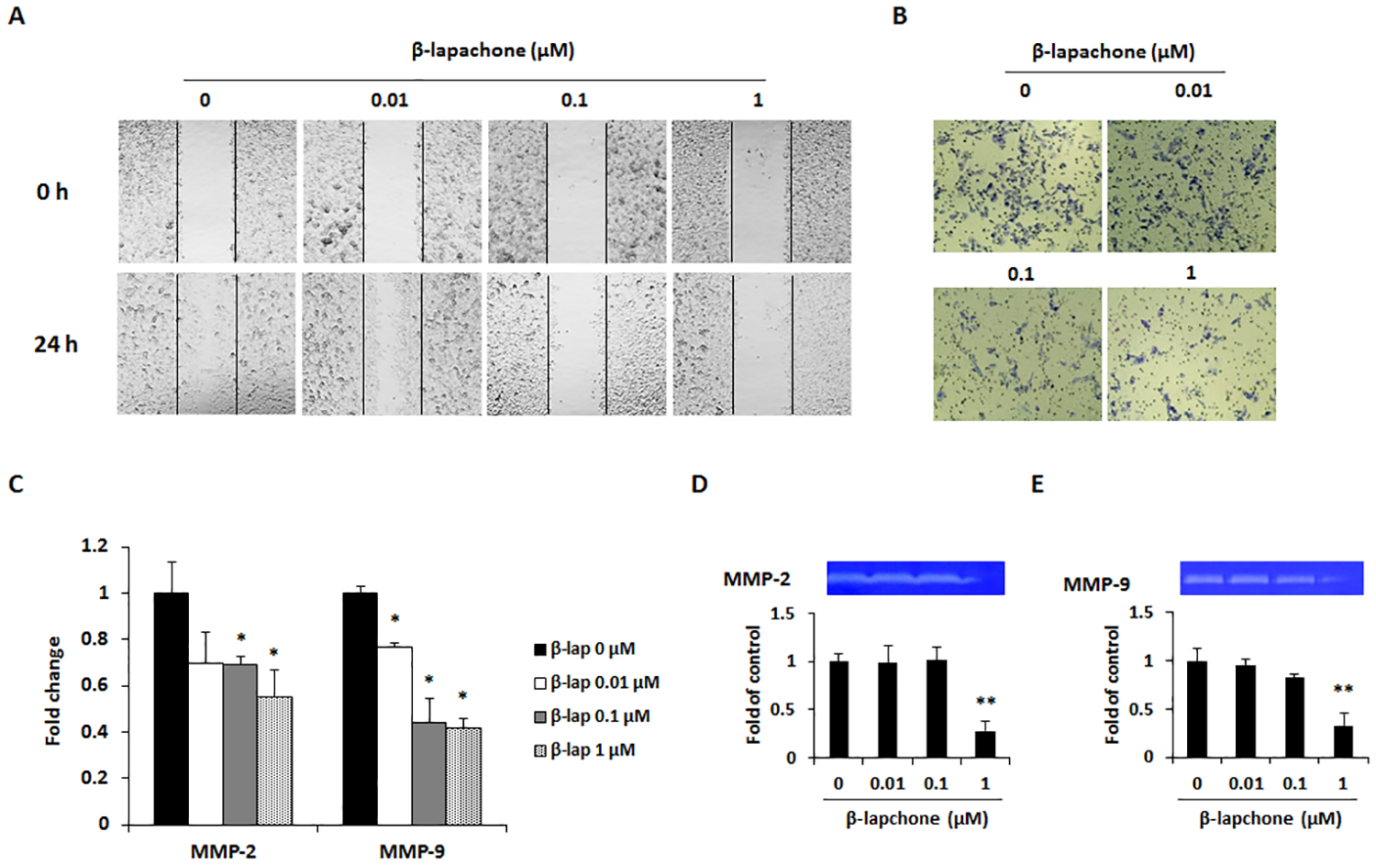
Effect of β-lapachone on the migration and invasion of B16F10 cells, through inhibition of MMP expression and activity. (A) Cancer cell motility was determined by wound healing assay. Cells were cultured to 8–90% confluent monolayer in a 6-well plate and scratched with a 200 μl micropipet tip. After washing with a serum-free medium, the cells were treated for 24 h with the indicated concentrations of β-lapachone. The denuded zone was observed at 0 and 24 h and images were photographed using a microscope (100× magnification). (B) Invasion ability of B16F10 cells was determined by invasion assay using matrigel-coated transwell chamber. (C) The mRNA expression levels of MMP-2 and MMP-9 were measured by real-time RT-PCR after β-lapachone treatment for 24 h. (D) Inhibitory effect of β-lapachone on activity of MMP-2 and MMP-9. After 12 h starvation, cells were treated with β-lapachone for 24 h and conditioned medium was collected for use in gelatin zymography. Photographs are representative of three independent experiments. The band intensity was quantitatively analyzed using ImageJ software. Values are the means ± SD of data from three independent experiments. *P < 0.05 and **P < 0.01.

To investigate the effect of β-lapachone on the migration and invasion of melanoma cells, a wound healing assay and invasion assay were performed. In the wound healing assay, control cells moved toward the scratched region, whereas β-lapachone dose-dependently suppressed migration of B16F10 cells (Fig 6A). On the other hand, the invasion ability of B16F10 cells was inhibited by various concentrations of β-lapachone (Fig 6B). Migration and invasion are typical features of cancer metastasis, and these features are associated with increased expression of MMPs. The enhanced MMP production is also one important factor involved in the EMT, migration, and invasion. Among the MMPs, MMP-2 and MMP-9 are the gelatinases expressed in cancer cells, and their proteolytic activity contributes to metastasis [24]. Therefore, we investigated the effect of β-lapachone on MMP-2 and MMP-9 expression and activity. As expected, β-lapachone inhibited the expression of MMP-2 and MMP-9 in B16F10 cells (Fig 6C). In addition, secretion of the gelatinolytic MMP-2 and MMP-9 was significantly decreased by β-lapachone treatment (Fig 6D and 6E). These results indicate that β-lapachone exerted an inhibitory effect on the migration and invasion ability of B16F10 cells by inhibiting MMP-2 and MMP-9 activity.

### β-Lapachone reduces EMT in B16F10 cells

**Fig 7:**
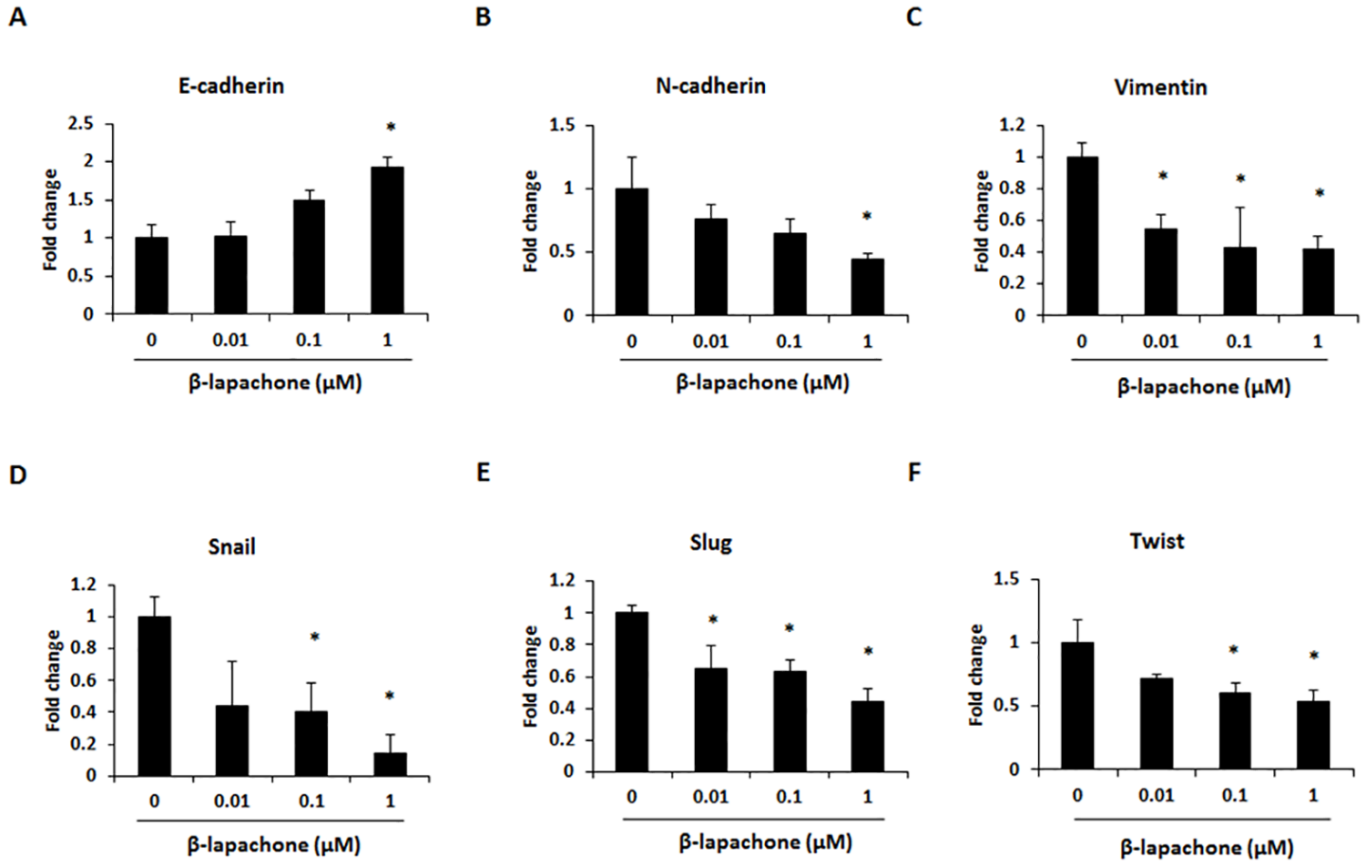
Effect of β-lapachone on the EMT marker expression in B16F10 cells. The mRNA expression levels of EMT-related genes were measured by real-time RT-PCR in β-lapachone-treated cells. B16F10 cells were treated with β-lapachone (0.01–1 μM) for 24 h. Values are the means ± SD of data from three independent experiments. *P < 0.05.

EMT-related gene expression was measured to elucidate the mechanisms underlying the inhibitory effect of β-lapachone on B16F10 cell migration and invasion. Our data showed that the mRNA expression of E-cadherin was significantly upregulated (Fig 7A), whereas the expression of N-cadherin, vimentin, snail, slug, and twist was downregulated in β-lapachone-treated B16F10 cells (Fig 7B–7F). These results further showed that the inhibitory effect of β-lapachone on B16F10 cell migration and invasion could be mediated through regulation of the EMT process.

### β-Lapachone reduces lung metastasis of B16F10 cells

**Fig 8:**
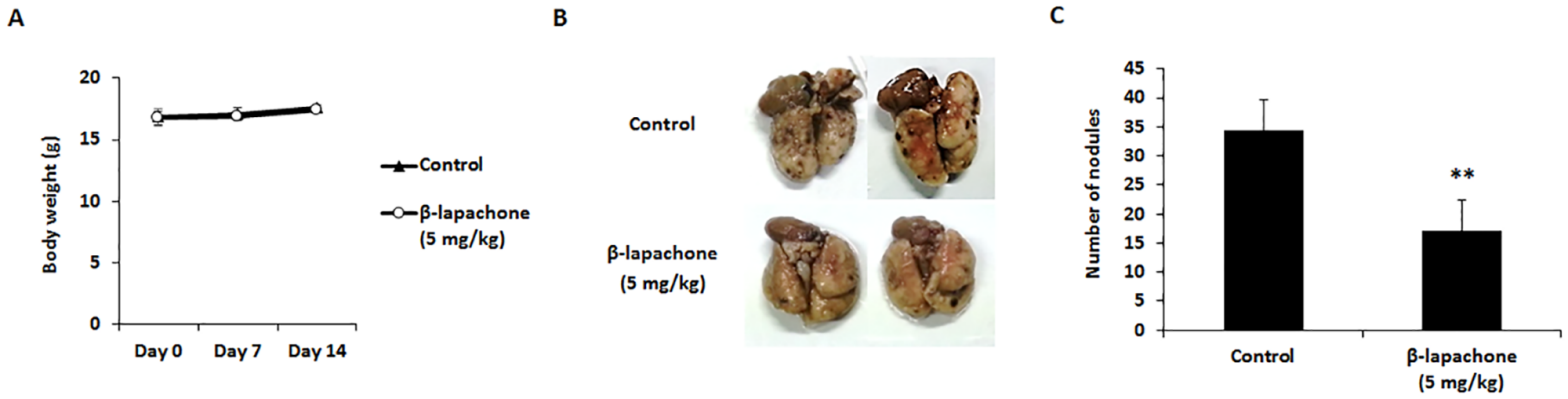
Effect of β-lapachone on the lung metastasis of B16F10 cells. C57BL/6 mice were intravenously injected with B16F10 cells (2 × 10^5^ cells) via the tail vein. The mice were intraperitoneally injected with DMSO or β-lapachone (5 mg/kg) every other day until sacrifice. (A) Measurement of body weight during β-lapachone administration. (B) Lungs were excised and fixed with 3.7% formaldehyde to count and compare the patterns of pulmonary tumor nodule formation among the experimental groups. (C) The number of tumor nodules is expressed as the mean ± SD. Values are the means ± SD of data from three independent experiments. **P < 0.01.

Based on the *in vitro* anti-metastatic potential of β-lapachone, the *in vivo* anti-metastatic effect of β-lapachone was tested in an experimental lung metastasis model. B16F10 cells were injected into the tail vein of C57BL/6 mice, and all mice were sacrificed on day 14 for evaluation. Intraperitoneal injections of 5 mg/kg β-lapachone or vehicle control (DMSO) were started 2 h before the melanoma cell injection, and continued to be administered every other day for 2 weeks. During the *in vivo* experiment, we measured body weight of mice to check significant toxicity of β-lapachone. Body weight after β-lapachone treatment did not show significant difference compared with control group (Fig 8A). As shown in Fig 8B and 8C, the number of metastatic lung nodules was significantly reduced in β-lapachone-treated mice as compared with the control group. This result proves that β-lapachone could suppress melanoma lung metastasis.

## Discussion

β-Lapachone, isolated from the lapacho tree, has been shown to exhibit anti-cancer activity in various types of cancer [16–19]. In particular, β-lapachone promotes cell cycle arrest and caspase-mediated apoptosis in human colon cancer cells [19]. These previous studies focused on apoptosis and cell cycle arrest in cancer cell lines. This study attempted to assess the anti-metastatic effects of β-lapachone on metastatic murine melanoma cell lines and in an experimental animal metastasis model. B16F10 and B16BL6 cells, which are derived from B16 mouse melanoma sublines, are normally employed to investigate the anti-metastatic effects of natural products [25, 26]. Bl6F10 cells were established from lung metastasis of B16 cells by i.v. injection through 10 successive selections [27]. B16BL6 cells were established from B16F10 cells collection from the penetrated mouse bladder wall [28]. Although these cells show similar characteristics, B16F10 cells are colonized in the lung by direct injection into the blood vessels, whereas subcutaneous injection of B16BL6 cells is spontaneously metastasized to the lung. The difference in the metastatic potential of these cells is due to the overexpression of LRF-1 in B16F10 cells [29]. Considering these characteristics, we investigated anti-metastatic effects of β-lapachone using both cell lines to increase the reliability.

Since cell cycle dysregulation is a hallmark of tumor cells, cell cycle arrest is another method of growth inhibition. The cell cycle is mediated by the activation of cyclin-dependent kinases (CDKs) through binding to specific cyclins. Therefore, modulation of cyclin and CDK expression is an important mechanism for inhibition of cell proliferation [30]. In several studies, β-lapachone showed an anti-tumor effect through induction of cell cycle arrest in colon and prostate cancer cells [19,31]. The present study demonstrated that G0/G1 phase arrest occurred in β-lapachone-treated B16F10 cells (Fig 2A and 2B). Furthermore, we carried out real-time RT-PCR to detect the effects of β-lapachone on the expression of cyclin and CDK.β-lapachone decreased the expression of cyclin D1 and CDK4, while cyclin D1-CDK4 complex inhibitor p16 expression was increased (Fig 2C). These results suggest that β-lapachone partly induced cell cycle arrest and that this effect is associated with downregulation of cyclin D1 and CDK4 expression. Although β-lapachone induced cell cycle arrest in B16F10 cells, G0/G1 phase rate was less than cell viability (Figs 1 and 2). It seems that G0/G1 phase arrest was occurred for the repair of DNA when β-lapachone treatment to B16F10 cells. However, failure of DNA repair leads to cell death of β-lapachone-treated cells. These results make us suppose that the decreased cell viability of B16F10 cells by β-lapachone treatment was due to mainly cell death.

Apoptosis is an essential mechanism for maintaining cellular homeostasis, as cancer occurs as a result of abnormal growth and uncontrolled proliferation of cells. Thus, induction of apoptosis in malignant cells is a major target of cancer therapy and metastasis treatment [32]. β-Lapachone treatment has been shown to completely inhibit cell survival and induce apoptosis in colon cancer cells [19]. Therefore, we first investigated cell viability in B16F10 and B16BL6 metastatic melanoma cells treated with various concentrations of β-lapachone. After β-lapachone treatment, cell viability of both B16F10 and B16BL6 cells decreased in a dose- and time-dependent manner (Fig 1). Using TUNEL assay and Annexin V assay, we proved that this inhibitory effect appeared to be mediated by the induction of apoptosis (Fig 2).

Apoptotic signals involve two main pathways which are extrinsic and intrinsic signaling pathway. In the extrinsic pathway, caspase-8 plays a central role in the transmission of the death signal by extracellular stimulation. The intrinsic pathway is regulated by anti-apoptotic (Bcl-2 and Bcl-xL) and pro-apoptotic (Bax) proteins of the Bcl-2 family. The balance in the expression levels of Bcl-2 family proteins is important for apoptosis [33]. Caspases are crucial effectors of apoptosis, as they cause proteolytic cleavage of many apoptosis-related proteins. Initiator caspases such as caspase-8 and caspase-9 are part of the extrinsic and intrinsic signaling pathways, respectively. Activation of these caspases results in apoptosis via activation of caspase-3 and cleavage of PARP, which is involved in DNA repair [34]. In the present study, β-lapachone reduced Bcl-2 and Bcl-xL expression, whereas Bax expression was increased in a dose- and time-dependent manner. Additionally, we found that cleavage of caspase-3, -8, and -9 and cleavage of PARP were induced by β-lapachone treatment (Fig 3A and 3B).

MAPKs, including p38, ERK, and JNK, are important mediators of signal transduction and are associated with a series of physiological processes such as cell proliferation, differentiation, and apoptosis [35]. Notably, MAPKs are implicated in both extrinsic and intrinsic apoptotic pathways, and are known to regulate apoptosis [36]. In a previous study, β-lapachone was found to induce apoptosis via activation of MAPK signaling pathways in human prostate cancer cell line DU145 cells [37]. Our data showed that β-lapachone increased phosphorylation of p38, ERK, and JNK (Fig 3C). Moreover, β-lapachone-induced inhibition of cell viability was significantly recovered by MAPK inhibitors (Fig 3D). These results suggest that β-lapachone-induced apoptosis is associated with both apoptotic pathways and MAPK signaling pathways in metastatic melanoma cells.

Metastasis is accompanied with various physiological changes including degradation of the ECM, over-expression of MMPs, the migration and invasion of cancer cells through the blood or lymphatic vessels, and subsequent transport to target organs [38]. We first explored the effects of β-lapachone on the metastatic ability of metastatic melanoma cells. β-Lapachone inhibited the migration and invasion of B16F10 cells in a dose-dependent manner (Fig 6A and 6B). MMPs have a critical function in metastasis; they cause ECM degradation and lead to cancer cells migrating to and invading distant organs [39]. In particular, MMP-2 and MMP-9 are known to degrade type IV collagen, which is the major component of the basement membrane, and induce cancer progression and metastasis [40]. To further explore the mechanisms of β-lapachone-induced inhibition of cell invasion and migration, we analyzed its effect on the expression and activity of MMP-2 and MMP-9 using real-time RT-PCR and gelatin zymography. We found that expression and activity of MMP-2 and MMP-9 were significantly reduced by treatment with β-lapachone (Fig 6C and 6D).

During metastasis, cancer cells frequently exhibit a loss of epithelial features and intercellular adhesion. This phenomenon is accompanied by increased cancer cell motility and acquisition of mesenchymal features. This process is called EMT, which is characterized by reduced expression of epithelial markers such as E-cadherin and increased expression of mesenchymal markers such as N-cadherin, vimentin, and snail. EMT regulates cell-cell adhesion and increases cancer cell motility and invasive properties [41]. In HepG2 and HepG3 hepatocarcinoma cells, β-lapachone shows an inhibitory effect on EMT by increasing E-cadherin expression and down-regulating snail [42]. In this study, the expression of EMT-related genes was analyzed to understand the anti-metastatic effect of β-lapachone on metastatic melanoma cells. We found that β-lapachone could regulate the EMT process by up-regulating E-cadherin and down-regulating N-cadherin, vimentin, snail, slug, and twist in B16F10 cells (Fig 7). Therefore, the inhibitory effect of β-lapachone on the migration and invasion of melanoma cells occurs through decreasing MMP activity and inhibiting the EMT process.

Although the anti-metastatic ability of β-lapachone through suppression of migration, invasion, and EMT in human hepatocarcinoma HepG2 cells was demonstrated [42], *in vivo* experimental evidence has not been reported. In this study, we first demonstrate the inhibitory effect of β-lapachone on metastatic melanoma with an *in vivo* model. We already established experimental lung metastasis mice model using B16F10 cells through preparatory experiment.

During the metastatic cascade, isolated tumor cells from a primary tumor are selected by undergoing microenvironment and obtained metastatic phenotypes to migrate and invade to distant organs. For these reasons, spontaneous metastasis model which is often subcutaneously inoculated displays similar process and results compared with clinical cases. However, spontaneous metastasis model shows several defectiveness including slow growth, low successful rate and inconsistent results [43]. On the other hand, experimental lung metastasis model, which is established by injection of the lateral tail vein in mice, is the most widespread experimental model [44]. It has several advantages for research such as short time course, consistent and reproducible result, and easy control of the number and type of cells. Especially, the B16F10 cells experience early stage of metastasis by re-injection for 10 times [27]. Therefore, experimental lung metastasis model by i.v. injection of B16F10 cells is proper to evaluate the anti-metastatic effect of β-lapachone on lung metastasis of melanoma cells. Body weight of mice, which is indicator of toxicity, did not change with injection of B16F10 cells (5 × 10^5^ cells) and the number of nodules in the lung was approximately 100–120. In this study, we injected B16F10 cells (2 × 10^5^ cells) to evaluate anti-metastatic effect of β-lapachone. β-Lapachone significantly decreased lung metastasis of melanoma in C57BL/6 mice (Fig 8). This anti-metastatic effect may result from *in vitro* effects of β-lapachone, such as the suppression of cell viability and decrease in metastatic abilities of B16F10 cells.

## Conclusions

In conclusion, the present study provides new evidence for the anti-metastatic effects of β-lapachone. Our results show that β-lapachone suppressed metastatic melanoma cell growth through induction of apoptosis and cell cycle arrest. β-Lapachone induced apoptosis by regulating the expression of caspases and Bcl-2 family proteins through the activation of p38, ERK, and JNK signaling pathways. Furthermore, β-lapachone inhibited the migration and invasion of B16F10 cells by increasing E-cadherin expression and decreasing expression of N-cadherin, vimentin, snail, slug, and twist. The *in vivo* experiment results show that β-lapachone can significantly reduce the number of tumor nodules and decrease lung weight. Based on these results, we conclude that β-lapachone may be a promising candidate in the search for agents that prevent metastatic melanoma.
